# Autoimmune Adrenalitis in Systemic Lupus Erythematosus: Identifying a Rare Endocrine Complication

**DOI:** 10.7759/cureus.96668

**Published:** 2025-11-12

**Authors:** Abdellatif Zhalka, Rami Jabareen, Mahmoud foqara, Karem Awad, Muhammad Zahlaka, Nizar Hijazi

**Affiliations:** 1 Endocrinology, Emek Medical Center, Afula, ISR; 2 Internal Medicine, Emek Medical Center, Afula, ISR; 3 Nephrology, Emek Medical Center, Afula, ISR; 4 Ear, Nose, and Throat (ENT), Wolfson Medical Center, Tel Aviv, ISR

**Keywords:** adrenalitis, antiphospholipid antibody syndrome (aps), clinical case, primary adrenal insufficiency, s: sle

## Abstract

Primary adrenal insufficiency (PAI) secondary to nonhemorrhagic adrenalitis is an extremely rare but potentially life-threatening endocrine manifestation in patients with systemic lupus erythematosus (SLE). We report the case of a 51-year-old man with longstanding SLE and antiphospholipid syndrome (APS) who presented with an adrenal crisis, characterized by fatigue, weight loss, vomiting, and abdominal pain. Laboratory evaluation revealed hypotension, metabolic acidosis, elevated adrenocorticotropic hormone (ACTH), and low serum cortisol, confirming the diagnosis of PAI. Abdominal CT imaging demonstrated bilateral adrenal gland enlargement with periadrenal fat stranding, consistent with nonhemorrhagic adrenalitis. The patient showed rapid clinical improvement following initiation of stress-dose corticosteroids and was subsequently discharged on lifelong glucocorticoid and mineralocorticoid replacement therapy.

This case emphasizes the importance of maintaining a high clinical suspicion for adrenal insufficiency in patients with SLE who present with nonspecific symptoms, such as fatigue, vomiting, and abdominal pain, that may be mistakenly attributed to a typical lupus flare, even when classic signs of adrenal hemorrhage or typical laboratory abnormalities are absent. Early diagnosis and treatment are critical to prevent life-threatening adrenal crises in this patient population.

## Introduction

Primary adrenal insufficiency (PAI) is a rare endocrine disorder characterized by impaired production of glucocorticoids and/or mineralocorticoids. The most common cause is autoimmune adrenalitis, frequently associated with autoimmune polyglandular syndromes involving type 1 diabetes, thyroiditis, or vitiligo. Other etiologies include adrenal hemorrhage, infections (e.g., tuberculosis), infiltrative diseases, and metastases [1].

Systemic lupus erythematosus (SLE) is a chronic multisystem autoimmune disease with diverse clinical manifestations. Adrenal involvement in SLE is rare and usually occurs in association with antiphospholipid syndrome (APS), a hypercoagulable state that predisposes to adrenal vein thrombosis and hemorrhagic infarction [2]. In contrast, nonhemorrhagic adrenalitis in SLE is exceedingly uncommon, with only a handful of reported cases [3,4]. The underlying mechanism remains uncertain but may involve immune-mediated destruction or microvascular ischemia rather than hemorrhage. Because adrenal symptoms frequently mimic lupus flares, manifesting as fatigue, hypotension, and electrolyte disturbances, recognition often occurs late in the disease course. Awareness of this rare presentation is critical, as timely diagnosis and corticosteroid replacement can be life-saving [5]. 

To our knowledge, this case represents one of the few reported instances of nonhemorrhagic adrenalitis in male patients with SLE and an uncommon manifestation in the context of APS, further highlighting the rarity of this presentation.

## Case presentation

A 51-year-old man with a 10-year history of SLE and triple-positive APS, including lupus anticoagulant (LA), elevated anticardiolipin IgG (75 GPL units; reference: <20), and anti-β₂ glycoprotein I IgG (60 SGU; reference: <20), with persistent positivity confirmed on repeat testing 12 weeks apart, was admitted with a one-week history of anorexia, fatigue, unintentional weight loss, generalized abdominal pain, and recurrent vomiting. His chronic medications included azathioprine, hydroxychloroquine, and warfarin. Notably, he had recently received rituximab infusions for lupus-related hematologic involvement. He denied fever, gastrointestinal bleeding, oral ulcers, or lymphadenopathy.

On physical examination, the patient appeared pale but was alert and oriented. Cardiopulmonary and abdominal examinations were unremarkable. Vital signs revealed a pulse of 90 beats per minute and blood pressure of 90/60 mmHg while supine, which dropped to 80/50 mmHg upon standing, suggestive of orthostatic hypotension.

Laboratory investigations (Table 1) showed mild anemia, lymphopenia, thrombocytopenia, and elevated blood urea nitrogen. Electrolytes revealed normonatremia and borderline hypokalemia. Arterial blood gas analysis demonstrated metabolic acidosis. Inflammatory markers were within normal limits. Coagulation studies showed an elevated international normalized ratio (INR), consistent with chronic warfarin use.

**Table 1:** Laboratory findings on admission BUN: blood urea nitrogen; ALT: alanine aminotransferase; AST: aspartate aminotransferase; PT: prothrombin time; INR: international normalized ratio; PTT: partial thromboplastin time

Test	Result	Reference range	Units
Complete blood count (CBC)			
White blood cell count (WBC)	11.99	4.0-10.0	x10⁹/L
Neutrophils (absolute)	11.00	2.0-7.5	x10⁹/L
Lymphocytes (absolute)	0.20	1.0-3.5	x10⁹/L
Eosinophils (absolute)	0.13	0.0-0.5	x10⁹/L
Hemoglobin (Hb)	10.8	13.5-17.5 (male)/12-16 (female)	g/dL
Mean corpuscular volume (MCV)	81.8	80-96	fL
Platelets	110	150-400	x10⁹/L
Chemistry panel			
Sodium (Na⁺)	136	135-145	mmol/L
Potassium (K⁺)	3.6	3.5-5.0	mmol/L
Chloride (Cl⁻)	106	98-107	mmol/L
Urea (BUN)	63	7-20	mg/dL
Creatinine	1.13	0.6-1.2	mg/dL
Glucose	108	70-100 (fasting)	mg/dL
AST (GOT)	21	10-40	U/L
ALT (GPT)	16	7-56	U/L
Total bilirubin	0.79	0.1-1.2	mg/dL
Venous blood gas (VBG)			
pH	7.32	7.31-7.41	-
pCO₂	37	41-51	mmHg
HCO₃⁻ (calculated)	19.1	22-28	mmol/L
pO₂ (venous)	48	30-50 (venous)	mmHg
Coagulation profile			
PT	33.9	11-13.5	sec
INR	2.9	0.9-1.1	-
PTT	54	25-35	sec
Inflammatory markers			
C-reactive protein (CRP)	0.4	<0.5	mg/dL

**Figure 1:**
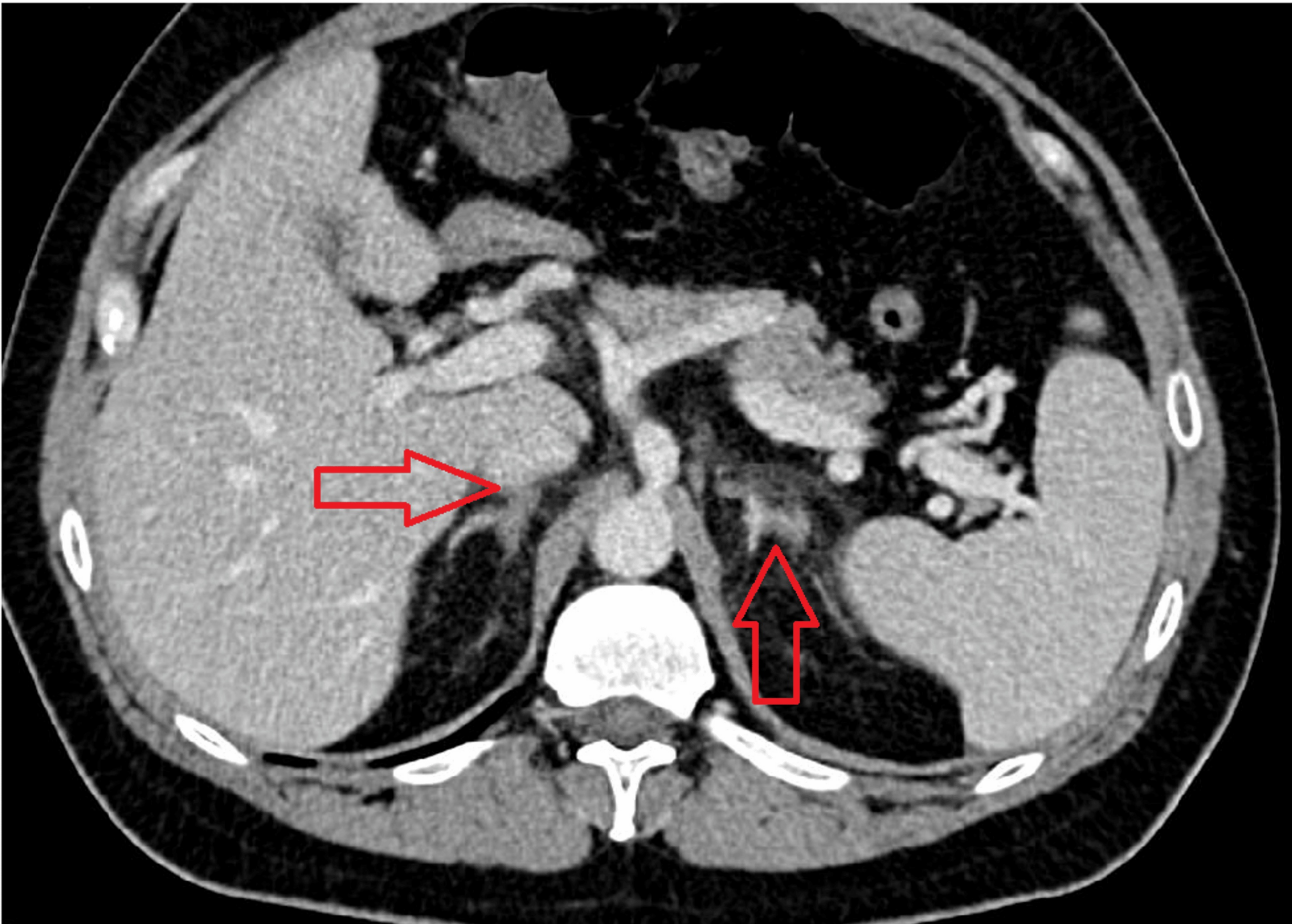
Axial contrast-enhanced CT image of the upper abdomen showing bilateral adrenal gland thickening with periadrenal fat stranding (arrows), consistent with nonhemorrhagic adrenalitis

**Figure 2:**
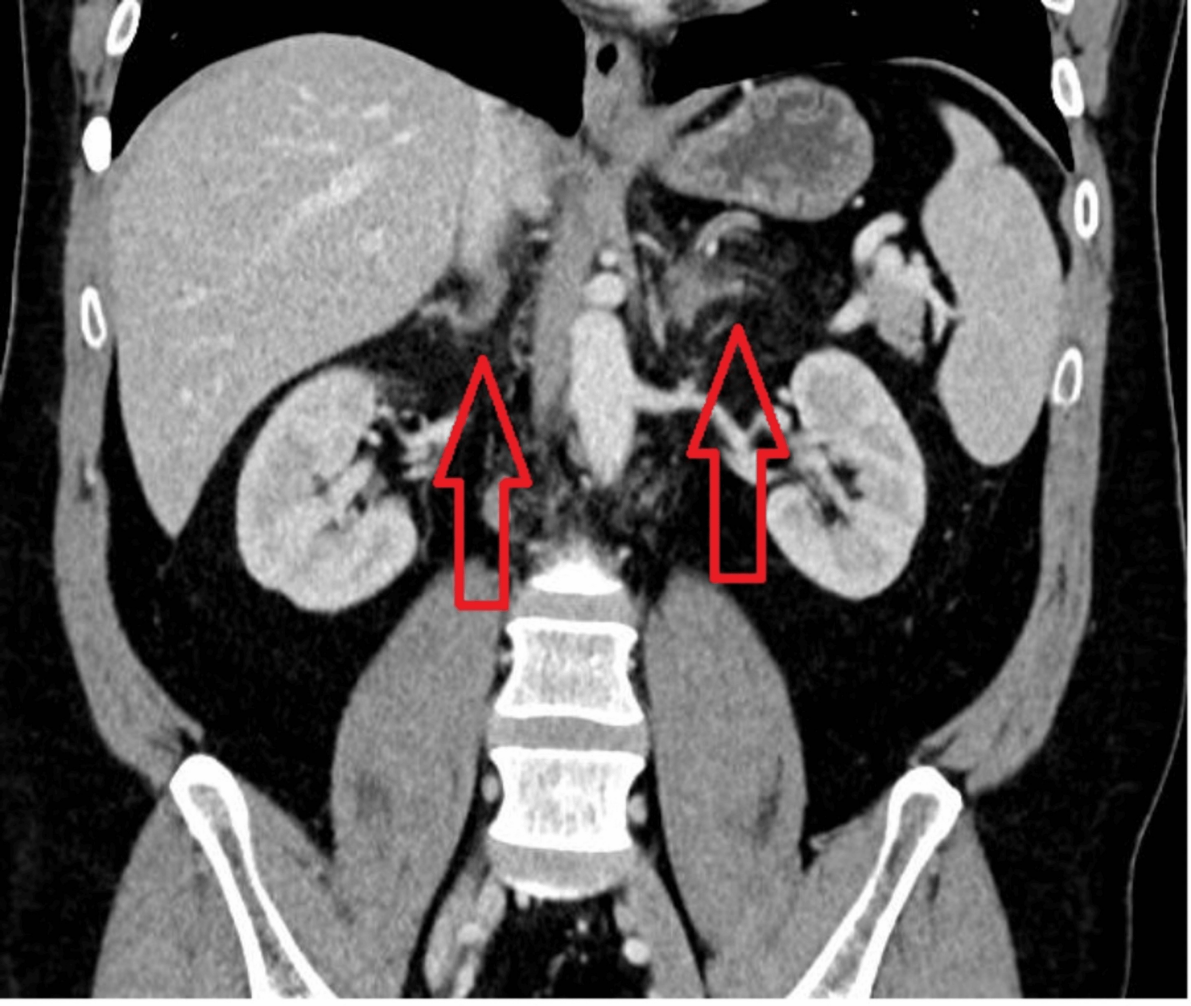
Coronal contrast-enhanced CT image demonstrating bilateral adrenal enlargement (arrows) and periadrenal fat stranding without evidence of hemorrhage

 Given the nonspecific gastrointestinal and constitutional symptoms alongside hypotension, a possible adrenal crisis was considered. An abdominal computed tomography (CT) scan (Figures 1, 2) revealed bilateral adrenal gland thickening with periadrenal fat stranding, without evidence of hemorrhage, findings suggestive of acute nonhemorrhagic adrenalitis.

In the context of known SLE and APS, PAI due to lupus-associated adrenalitis was suspected. Empiric stress-dose intravenous hydrocortisone therapy was initiated, leading to rapid clinical improvement and stabilization of blood pressure.

Subsequent hormonal evaluation (Table 2) showed a morning serum cortisol level of <5.1 µg/dL and a markedly elevated ACTH level. This finding favored an acute rather than chronic process, as acute adrenal insufficiency typically presents with sudden hemodynamic instability, whereas chronic PAI is characterized by long-standing ACTH elevation, hyperpigmentation, and persistent electrolyte abnormalities-features absent in our patient.

**Table 2:** Hormonal evaluation ACTH: adrenocorticotropic hormone

Test	Result	Reference range	Units
Morning serum cortisol	3.2	6.7-22.6	µg/dL
ACTH	220	7.2-63.3	pg/mL
Synacthen test-30 min	8	>18	µg/dL
Synacthen test-60 min	10	>18	µg/dL
Thyroid-stimulating hormone (TSH)	2.3	0.4-4.5	mIU/L
Free T4	1.2	0.8-1.8	ng/dL

After clinical stabilization and improvement in laboratory parameters, the patient was discharged on lifelong oral cortisone acetate and fludrocortisone. Two days later, a short Synacthen (ACTH stimulation) test was performed after temporarily withholding cortisone acetate and fludrocortisone for 24 hours. The test demonstrated a suboptimal cortisol response (30-minute: 8 µg/dL; 60-minute: 10 µg/dL), confirming the diagnosis of PAI. Thyroid function tests indicated subclinical hypothyroidism with negative anti-thyroid peroxidase (anti-TPO) antibodies, excluding autoimmune polyglandular syndrome. At outpatient follow-up, he remained clinically stable with no recurrence of symptoms.

## Discussion

Adrenal involvement in SLE and APS is uncommon and usually presents as hemorrhagic adrenalitis secondary to APS-related thrombosis. PAI occurs in approximately 0.4% of APS patients over a five-year follow-up [6], but the incidence rises to 10-26% in catastrophic APS [7]. Adrenalitis in this setting can be classified into two types: the more common hemorrhagic form and the rare nonhemorrhagic form, which has only been reported in a few cases in the literature [3].

Adrenalitis in autoimmune conditions results from either vascular or immune-mediated injury to the adrenal glands. In APS, a hypercoagulable state can cause thrombosis of the adrenal veins, leading to infarction, hemorrhage, and ischemic damage [2]. In SLE, autoimmune mechanisms can directly target the adrenal cortex, often involving antibodies against steroidogenic enzymes such as 21-hydroxylase, resulting in PAI [8]. Although both mechanisms are rare, they highlight the importance of recognizing adrenal involvement in patients with SLE and APS.

In clinical practice, SLE-associated adrenalitis often manifests with nonspecific symptoms such as fatigue, abdominal pain, vomiting, nausea, lethargy, and hyperpigmentation. Laboratory evaluation commonly reveals low cortisol levels, elevated ACTH, hyponatremia, and hyperkalemia [9-11]. Early recognition of these signs is essential to prevent adrenal crises. For clarity, the key clinical and laboratory findings summarized in Table 3 are based on the study by Espinosa et al. [11].

**Table 3:** The most common clinical and biochemical features of adrenal involvement in SLE and APS, identifying abdominal pain and hyponatremia as the leading clinical symptom and laboratory abnormality, respectively, in patients with SLE-associated adrenalitis, according to Espinosa et al. [11] ACTH: adrenocorticotropic hormone; SLE: systemic lupus erythematosus; APS: antiphospholipid syndrome

Finding	n (%)
Abdominal pain	47 (55%)
Hypotension	46 (54%)
Fever	34 (40%)
Nausea or vomiting	27 (31%)
Weakness or fatigue	27 (31%)
Lethargy/altered mental status	16 (19%)
Hyponatremia	50 (58%)
Hyperkalemia	39 (45%)
Adrenal hemorrhage (imaging)	49 (57%)
Hemorrhagic infarction (histology)	47 (55%)
Lupus anticoagulant positive	83 (97%)
Anticardiolipin antibody positive	80 (93%)
Decreased baseline cortisol	84 (98%)
Increased ACTH	83 (96%)
Cosyntropin test positive	86 (100%)

Histopathological examination remains the definitive method to diagnose adrenal involvement. APS-related adrenal hemorrhage typically shows hemorrhagic infarction and tissue necrosis, while SLE-associated adrenalitis reflects autoimmune destruction of the adrenal cortex, causing PAI [9,10]. However, adrenal biopsy is rarely performed due to the retroperitoneal location and risk of exacerbating damage. Thus, imaging modalities such as CT and MRI combined with laboratory findings usually suffice for diagnosis [9]. The ACTH stimulation test remains the gold standard for confirming PAI [12].

Table 4 highlights previously reported cases of adrenalitis in SLE, predominantly affecting female patients with hemorrhagic adrenalitis. To our knowledge, this represents one of the few reported male patients with nonhemorrhagic adrenalitis associated with SLE, emphasizing the importance of considering adrenal insufficiency in all SLE patients presenting with nonspecific symptoms.

**Table 4:** Clinical features and outcomes of adrenal involvement in SLE and APS: a literature summary SLE: systemic lupus erythematosus; APS: antiphospholipid syndrome; LMWH: low molecular weight heparin

No.	Source	Age (years)/gender	Diagnosis	Clinical presentation	Type of adrenal involvement	Treatment	Prognosis
1	Liang et al.(2024) [3]	55/female	Adrenal infarction with SLE and APS	Abdominal pain, vomiting, hypotension	Nonhemorrhagic	Methylprednisolone, hydroxychloroquine, LMWH	Recovery: stable at 1 year
2	Bhat et al. (2014) [13]	20/female	Autoimmune adrenalitis in SLE	Hyperpigmentation, fatigue, weight loss	Nonhemorrhagic	Prednisolone	Remission
3	Batt et al. (2016) [14]	40/female	Bilateral adrenal infarction in APS + SLE	RUQ pain, vomiting, fever	Nonhemorrhagic	Steroids, anticoagulation	Recovered
4	Jiang et al. (2023) [15]	32/female	Bilateral adrenal hemorrhage in SLE + APS	Abdominal pain, hyperpigmentation	Hemorrhagic	Corticosteroids	Full recovery

A limitation of our report is the absence of an adrenal biopsy; however, due to its invasiveness and the strong clinical and radiologic evidence, a biopsy was not performed. This case emphasizes the importance of clinicians to consider adrenal involvement in SLE patients regardless of traditional hemorrhagic presentation or laboratory abnormalities. Our patient presented with abdominal pain, nausea, vomiting, and orthostatic hypotension but showed no electrolyte imbalances. In the context of SLE and APS, along with imaging findings, we diagnosed adrenal insufficiency. Early recognition allows for prompt corticosteroid therapy, which can prevent adrenal crises and long-term hormone deficiencies. This case broadens current understanding by showing that adrenal involvement in SLE can occur without typical lab changes and can affect males as well. Future research should aim to develop screening protocols for adrenal involvement in SLE and APS to improve detection and patient outcomes.

## Conclusions

Adrenal involvement in SLE and APS, though rare, can lead to life-threatening PAI. Our case, the first reported male patient with nonhemorrhagic adrenalitis in SLE, highlights the importance of maintaining a high clinical suspicion even in the absence of classic hemorrhagic changes or typical laboratory abnormalities. Early recognition and timely corticosteroid treatment are pivotal to preventing adrenal crises and improving patient outcomes. This report expands current understanding of adrenal complications in autoimmune diseases and highlights the importance of considering adrenal evaluation in all SLE patients presenting with nonspecific symptoms. Further studies are warranted to guide screening and management strategies for adrenal involvement in this population.
